# Latent profile analysis and associated factors of academic motivation among master's nursing students: a cross-sectional study

**DOI:** 10.3389/fmed.2026.1962934

**Published:** 2026-10-14

**Authors:** Kaiwen Zhan, Lei Liu, Jiayu Wang, Yingjie Wang, Manman Li, Yu Tian, Huijuan Tong

**Affiliations:** 1 School of Nursing, Liaoning University of Traditional Chinese Medicine, Shenyang, China; 2 Department of Nursing, The Affiliated Hospital of Xuzhou Medical University, Xuzhou, China; 3 Department of Nursing, The First Affiliated Hospital of Liaoning University of Traditional Chinese Medicine, Shenyang, China; 4 Department of Nursing, Liaoning Cancer Hospital & Institute, Shenyang, China; 5 School of Nursing, Shenyang Medical College, Shenyang, China

**Keywords:** academic motivation, autonomy support, basic psychological needs, latent profile analysis, master's nursing students, self-determination theory

## Abstract

**Background:**

Academic motivation is a critical factor for ensuring the training quality of master's nursing students. Affected by individual backgrounds and psychological characteristics, academic motivation within this group exhibits heterogeneity. This study adopted latent profile analysis to identify the latent classes of academic motivation among master's nursing students and analyze the factors associated with each class.

**Methods:**

A cross-sectional survey was conducted among 390 master's nursing students from 15 universities in China between November 2024 and February 2025. The sociodemographic questionnaire, Academic Self-Regulation Questionnaire, Learning Climate Questionnaire and Basic Psychological Need Satisfaction Scale were adopted for data collection. Latent profile analysis was used to classify the academic motivation among master's nursing student, and the R3STEP method was employed to conduct multinomial logistic regression analysis to explore factors associated with each class.

**Results:**

The academic motivation among master's nursing students could be divided into four latent classes: ‘relatively low motivation’ group (40.3%), ‘relatively high autonomous motivation’ group (23.3%), ‘relatively high controlled motivation’ group (19.7%) and ‘relatively high motivation’ group (16.7%). Compared with the ‘relatively low motivation’ group, older age was associated with a lower likelihood of belonging to the ‘relatively high motivation’ group. Students who were female, who chose nursing as their first choice, and who perceived higher autonomy support and greater satisfaction of basic psychological needs were more likely to belong to the ‘relatively high autonomous motivation’ group. Students who chose nursing as their first choice and who perceived higher autonomy support were more likely to fall into the ‘relatively high motivation’ group.

**Conclusions:**

The academic motivation among master's nursing students is heterogeneous. Autonomy-supportive learning environments and attention to students’ basic psychological needs may represent relevant areas for educational support and future intervention research.

## 1 Introduction

Master's education in nursing has become a key initiative for enhancing the quality of the global nursing workforce and improving nursing service standards (1, 2). Approximately 50 nations worldwide have established clinically oriented master's degree programs in nursing designed to train advanced practice nurses (APNs) (3). China established the Master of Science in Nursing (MSN) degree in 1990 with the aim of training nurse educators, researchers, and administrators (1). In order to train highly qualified nursing professionals, the Academic Degrees Committee of the State Council announced the establishment of the Master of Nursing Specialist (MNS) degree in 2010 (4). With the gradual expansion of enrollment scales for masters' nursing students in China, this cohort has become core promoters of disciplinary innovation in nursing and clinical nursing reform (5). Against the rapid development of the healthcare industry, self-directed learning is an essential approach for masters' nursing students to keep pace with disciplinary frontiers (6). Academic motivation constitutes the primary psychological driver for maintaining self-directed learning among individuals (7). Academic motivation refers to an internal psychological state that arouses and sustains an individual's learning activities, directing such activities toward specific learning goals, and satisfies certain learning needs (8). Academic motivation can encourage students to engage in learning (9), facilitate their self-directed learning (10), and achieve academic well-being (11). Accordingly, academic motivation directly affects the training outcomes of high-level nursing talents.

As one of the core theories in the field of motivation research, Self-determination theory (SDT) explicitly proposes that the quality of motivation matters more than its quantity (12). According to the level of motivation autonomy, it classifies motivation into autonomous motivation and controlled motivation (12). Autonomous motivation refers to the motivation to take action voluntarily driven by personal volition and interest, whereas controlled motivation refers to the motivation to act compelled by internal or external pressure (12). Research has confirmed that autonomous motivation is generally associated with a broader and more enduring spectrum of positive outcomes relative to controlled motivation. Students with higher autonomous motivation tend to demonstrate more positive learning behaviors, including devoting more time to study, adopting deep learning strategies, and attaining superior academic performance (13). Research indicates that the overall academic motivation of graduate nursing students is not yet ideal, and there is a tendency for their autonomous motivation to decline over time. El-Sayed et al. (14) conducted a questionnaire survey among 410 graduate nursing students in Egypt, and the results indicated that students' academic motivation was at a moderate level. Bingen et al. (15) conducted a longitudinal study on the changes in the academic motivation of 17 advanced practice nursing students. The results revealed that students' autonomous motivation declined significantly after the first semester, and their academic motivation shifted from autonomous motivation to controlled motivation (15).

Both of the above studies adopted a variable-centered approach, which ignored the heterogeneity among individuals and was unable to precisely distinguish different motivation types within the population of master's nursing students. Compared with the traditional variable-centered approach, the individual-centered approach can focus on individual differences in motivation and accurately identify latent classes within the population. Vansteenkiste et al. (16) classified college students' academic motivation into four classes based on SDT: a good quality motivation group, a poor quality motivation group, a low quantity motivation group, and a high quantity motivation group, which provides empirical references for the present study. Latent profile analysis (LPA), as an individual-centered analytical method, can classify individuals according to their different response patterns on explicit variables and divides individuals with similar response patterns into the same latent class, making intra-class differences as small as possible and inter-class difference as large as possible (17). Therefore, employing LPA to explore latent classes of academic motivation among master's nursing students can effectively address the limitations of existing studies and offer a scientific foundation for implementing targeted academic motivation interventions of master's nursing students.

Previous studies have shown that, within the framework of SDT, the factors associated with academic motivation mainly include psychological mechanism and contextual factors. SDT posits that satisfaction of the three basic psychological needs including autonomy, competence, and relatedness represents a psychological mechanism closely associated with individuals' autonomous motivation (18). Furthermore, SDT emphasizes that autonomy support, as a critical contextual factor, correlates with individuals' autonomous motivation (19). Studies indicate that higher perceived autonomy support among students tends to coincide with greater satisfaction of basic psychological needs, accompanied by elevated autonomous motivation, increased study engagement, and the adoption of deeper learning strategies (20). In addition, sociodemographic factors such as age (21), gender (22), grades (23), work experience (24) also have associations with academic motivation. Therefore, an in-depth understanding of the associations between basic psychological needs and autonomy support within the SDT framework on academic motivation, combined with the sociodemographic characteristics of master's nursing students, carries important implications for the subsequent improvement of educational environments and the development of intervention strategies.

Hence, this study aimed to identify the latent classes of academic motivation among master's nursing students through LPA, and to analyze the associated factors of each latent class, including sociodemographic characteristics, autonomy support, and basic psychological needs.

## 2 Methods

### 2.1 Study design

This cross-sectional study was conducted and reported in accordance with the STROBE guidelines of the EQUATOR Network (25, 26).

### 2.2 Participants

This study adopted a convenience sampling method. Drawing on China's seven major geographical regions (*N*ortheast, East, North, Central, South, Southwest, and Northwest), and taking into account the distribution of nursing master's degree-awarding programs, graduate enrollment size, and regional representativeness within each region, two to three universities cultivating master's nursing students were selected from each region, yielding a total of 15 universities. Inclusion criteria were currently enrolled master's nursing students. Exclusion criteria were leave of absence or study abroad. According to the Kendall multifactor analysis sample size estimation method, the sample size is generally 5–10 times the number of variables (27). A total of 15 variables were included in this study, and 20% of the invalid questionnaires were taken into account. The sample size required for this study was calculated to be 90–180 cases. Combined with the recommended minimum sample size of 300–500 for LPA (28), 420 master's nursing students were invited to participate in the survey. In total, 420 questionnaires were distributed and 401 were returned. After excluding 11 questionnaires with identical responses to 10 or more consecutive items, 390 valid questionnaires were retained, with an effective response rate of 97.3%.

### 2.3 Measures

#### 2.3.1 Sociodemographic characteristics

By reviewing the relevant literature and designing their own according to the needs of the study, including age, gender, nationality, grades, undergraduate training process, nursing as first choice or not, in-service or not.

#### 2.3.2 Academic motivation

The Academic Self-Regulation Questionnaire (SRQ-A), originally developed by Ryan and Connell (29) and translated into Chinese by Bao (30), was used to assess academic motivation among master's nursing students. The SRQ-A comprises 30 items across four dimensions: external regulation (11items), introjected regulation (5 items), identified regulation (7 items), and intrinsic motivation (7 items). Items are rated on a 5-point Likert scale from 1 (completely inconsistent) to 5 (completely consistent). The questionnaire offers three scoring methods. First, computing the mean score for each dimension yields four motivation scores reflecting varying degrees of internalization. Second, averaging external and introjected regulation scores provides an indicator of controlled regulation, whereas averaging identified regulation and intrinsic motivation scores indicates autonomous regulation. Controlled regulation reflects less internalized motivation and autonomous regulation reflects more internalized motivation. Third, the Relative Autonomy Index (RAI) is calculated as a weighted sum: RAI = 2  ×  intrinsic motivatio*n* + identified regulation−introjected regulation−2 × external regulation. The RAI indicates the extent to which an individual's academic motivation tends toward autonomy; higher scores reflect greater autonomous motivation, whereas lower scores indicate greater controlled motivation. Bao (30) measured the Cronbach's alpha coefficient for the total questionnaire to be 0.7934 and for each of the four dimensions to be 0.7982 to 0.8536 among 1,559 junior high school students.

#### 2.3.3 Autonomy support

The Learning Climate Questionnaire (LCQ), developed by Williams and Deci (31) and translated into Chinese by Chen (32), assessed perceived teacher autonomy support among master's nursing students. This unidimensional 15-item scale uses a 5-point Likert scale (1 = completely inconsistent to 5 = completely consistent), with item 13 reverse-scored. Higher average scores indicate greater perceived autonomy support. Chen (32) applied the LCQ to high school students and measured the Cronbach's alpha coefficient to be 0.876.

#### 2.3.4 Basic psychological needs

The Basic Psychological Need Satisfaction Scale (BPNSS), developed by Gagné (33) and translated into Chinese by Li (34), assessed basic psychological needs satisfaction. This 21-item scale covers three dimensions: autonomy (7 items), competence (6 items), and relatedness (8 items). Items are rated on a 7-point Likert scale (1–7), with nine items (3, 4, 7, 11, 15, 16, 18, 19, and 20) reverse-scored. Total scores range from 21 to 147, with higher scores indicating greater need satisfaction. Li (34) measured the Cronbach's alpha coefficient for the total scale to be 0.842 among 362 high school students.

### 2.4 Data collection

A questionnaire survey was conducted among master's nursing students who met the inclusion and exclusion criteria from November 2024 to February 2025, with the assistance of teachers from school of nursing of the university. Before the formal survey, all investigators received uniform training to familiarize themselves with the questionnaire and to provide guidance when needed. They then screened participants strictly according to the inclusion and exclusion criteria. Prior to questionnaire administration, the investigators informed the participants of the purpose and significance of this study. After giving their consent, participants signed the informed consent forms.

We collected data through both online and offline methods. For online collection, we used Questionnaire Star (https://www.wjx.cn/), a Chinese platform, and distributed the questionnaire via WeChat using QR codes. Each IP address was limited to one response, and all items were required before submission. For offline collection, we administered paper questionnaires face-to-face, with participants completing them independently. The combination of mandatory item settings on the online platform and on-site supplementary completion for paper questionnaires guaranteed item completeness. Thus, no missing data was observed in all returned questionnaires. All raw data are securely encrypted and stored by dedicated personnel. Data are de-identified before analysis, and all final outcomes are reported only as aggregates to prevent disclosure of participants' personal information.

### 2.5 Data analyses

The SRQ-A, LCQ and BPNSS have been translated and validated among middle school students, whereas the participants in the present study were master's nursing students, who differ from middle school students in educational stage and professional background. According to the International Test Commission, psychometric properties should be re-examined when scales are administered in a new target population (35). Prior to formal analyses, SPSS 25.0 was used to calculate Cronbach's alpha coefficients of each scale to evaluate internal consistency reliability; an alpha value ≥0.70 was considered acceptable (36). Subsequently, confirmatory factor analysis (CFA) was performed for each scale using AMOS 24.0 to examine construct validity. Model fit criteria were based on recommendations from Hu and Bentler, and Wu: *χ*^2^/df < 3, CFI and TLI > 0.95, RMSEA < 0.06, SRMR < 0.08 (37, 38).

Descriptive statistical analyses were performed using SPSS 25.0 to characterize the distribution of sociodemographic characteristics, including age, gender, nationality, grades, undergraduate training process and other variables. Continuous variables were reported as means and standard deviations. Categorical variables were expressed as absolute values and percentages.

The 30 item scores of the SRQ-A were taken as observed variables, and LPA was performed using Mplus 8.3. Likert 5-point items were treated as continuous variables, and the maximum likelihood estimation method was adopted for parameter estimation. Residual variances and covariances within each class were constrained to be equal across classes. Missing values were handled automatically via the full-information maximum likelihood method. During model estimation, 1,300 sets of random starting values were specified, and the best 250 iterations were retained for final optimization to ensure that the log-likelihood value converged to the global optimal solution. Instead of using total scores of the four dimensions directly, the present study utilized all 30 items, aiming to fully retain heterogeneous information from individuals' micro-level item responses and avoid loss of inherent variability caused by dimension-wise average scores, so as to improve the sensitivity of class identification. Model fit was evaluated based on the Akaike Information Criterion (AIC), Bayesian Information Criterion (BIC), Sample-size Adjusted BIC (aBIC), Lo-Mendell-Rubin Likelihood Ratio Test (LMR), Bootstrapped Likelihood Ratio Test (BLRT), and entropy (17). In general, lower values of AIC, BIC, and aBIC indicate better model fit. A significant *p*-value (< 0.05) for LMR and BLRT suggests that the K_0_-class model exhibits better model fit than the K_−1_-class model. Entropy ranges from 0 to 1, with values closer to 1 indicating more accurate classification. Referring to the methodological recommendations of Spurk et al. (39) and Sorgente et al. (40), this study further set 2,000 random starting values and retained the best 500 iterations for sensitivity analysis. The robustness of classification results was evaluated by comparing the replication frequency of the optimal log-likelihood values and the proportion of participants in each class under the two sets of random starting schemes.

Prior to the multinomial logistic regression analysis, the variance inflation factor (VIF) was used to test multicollinearity among independent variables. A VIF value greater than 5 was defined as indicative of significant multicollinearity in the present study. The VIF values of all independent variables ranged from 1.023 to 1.567, suggesting no evident multicollinearity. Subsequently, the R3STEP method in Mplus 8.3 was used to conduct multinomial logistic regression analysis to explore factors associated with latent classes of academic motivation among master's nursing students. The R3STEP method allows estimation of associations between independent variables and latent class membership while accounting for classification error, and is more robust than the traditional classify-analyze strategy (41). A two-sided *p*-value of less than 0.05 was considered statistically significant. This study reports the complete results of the multinomial logistic regression, including unstandardized coefficient (*β*), standard error (SE), Wald χ^2^, odds ratio (OR), 95% confidence interval (95% CI), exact *p*-values, to facilitate interpretation and enhance reproducibility (42).

### 2.6 Ethical considerations

This study was approved by the Ethics Committee of Shenyang Medical College [(2024) Ethics Review No. (5)], and all procedures were performed in accordance with the Declaration of Helsinki. Informed consent was obtained from participants before data collection. The data will only be used for the present study and will not be provided to third parties or utilized in other subsequent research.

## 3 Results

### 3.1 Reliability and validity of research instruments

In this study, the overall Cronbach's alpha coefficient of the SRQ-A was 0.864, and the Cronbach's alpha coefficients for its four dimensions ranged from 0.760 to 0.887. The Cronbach's alpha coefficient of the LCQ was 0.961. The overall Cronbach's alpha coefficient of the BPNSS was 0.851. The internal consistency reliability of all scales reached an acceptable level. CFA was performed separately for the SRQ-A, LCQ, and BPNSS. The fit indices of the four-factor model for the SRQ-A were: *χ*^2^/df = 3.893, CFI = 0.802, TLI = 0.785, RMSEA = 0.086, SRMR = 0.108; the fit indices of the one-factor model for the LCQ were: *χ*^2^/df = 6.306, CFI = 0.928, TLI = 0.916, RMSEA = 0.117, SRMR = 0.036; the fit indices of the three-factor model for the BPNSS were: *χ*^2^/df = 10.093, CFI = 0.530, TLI = 0.470, RMSEA = 0.153, SRMR = 0.515.

### 3.2 Participant characteristics

The mean age of the participants was 24.8 ± 2.9. The majority of participants were female (86.2%), Han Chinese (93.3%), nursing as first choice (74.1%), not in-service (91.8%). Complete participant characteristics are shown in Table 1.

**Table 1:** Participants’ sociodemographic characteristics (*N* = 390).

Variables	M ± SD	*n* (%)
Age	24.8 ± 2.9	
Gender		
Female		336 (86.2)
Male		54 (13.8)
Nationality		
Han nationality		364 (93.3)
Others		26 (6.7)
**Grades**		
2021		66 (16.9)
2022		125 (32.1)
2023		199 (51.0)
Undergraduate training process		
Technical secondary school to undergraduate and college to undergraduate		103 (26.4)
General undergraduate		287 (73.6)
Nursing as first choice		
Yes		289 (74.1)
No		101 (25.9)
In-service		
Yes		32 (8.2)
No		358 (91.8)

### 3.3 LPA results for academic motivation

The 30 item scores of the SRQ-A were used as observed variables in this study, and 1–5 models were fitted sequentially. Model fit indices are presented in Table 2. The results showed that AIC, BIC and aBIC decreased as the number of latent classes increased, indicating that increasing the number of classes could improve model fit to a certain extent. The Entropy values of all models were greater than 0.9, suggesting high classification accuracy. The BLRT tests for all models were significant (*p* < 0.05), meaning that each additional class yielded a statistically significant improvement in model fit compared with the preceding model. The LMR test for Model 3 was significant (*p* < 0.05), whereas the LMR tests for Model 4 and Model 5 were non-significant (*p* > 0.05), demonstrating that Model 4 and Model 5 did not significantly improve model fit relative to Model 3. Nevertheless, further examination of the class characteristics of candidate models revealed that Model 4 extracted a ‘relatively high motivation’ group with high autonomous motivation and high controlled motivation from Model 3. This group accounted for 16.7% of the sample and represented a distinctive integrated state with coexisting autonomous and controlled motivation within the SDT framework, possessing independent theoretical implications and practical value. The proportion of participants across the four classes in Model 4 were 40.3%, 23.3%, 19.7%, and 16.7%, all above 5%. The average posterior probabilities ranged from 0.936 to 0.990, indicating reliable classification quality. In contrast, the new class in Model 5 are merely a further subdivision of the ‘relatively low motivation’ group in Model 4, lacking an independent theoretical explanatory logic and having limited practical significance. The sensitivity analysis results suggest that Model 4's optimal log-likelihood value can be stably reproduced under two sets of random starting configurations, with consistent sample proportions across groups, indicating a robust classification structure. Taking into account the model fit indices, class interpretability, and theoretical significance, this study ultimately selected Model 4 for subsequent data analysis. The class characteristic plot for Model 4 is presented in Figure 1, while those for Models 3 and 5 are provided in the Supplementary Materials.

**Table 2:** The LPA model fit indices.

Model	AIC	BIC	aBIC	LMR		BLRT		Entropy	Category probability
				*p*-value		*p*-value			
Model 1	35,817.331	36,055.300	35,864.923	–		–	–		1.000
Model 2	34,343.570	34,704.490	34,415.752	<0.001		<0.001	0.927		0.633/0.367
Model 3	33,274.771	33,758.641	33,371.542	<0.001		<0.001	0.925		0.379/0.318/0.303
Model 4	32,878.856	33,485.677	33,000.217	0.240		<0.001	0.936		0.403/0.233/0.197/0.167
Model 5	32,529.952	33,259.723	32,675.902	0.391		<0.001	0.941		0.092/0.241/0.392/0.154/0.121

AIC, Akaike information criterion; BIC, Bayesian information criterion; aBIC, Sample-size adjusted BIC; LMR, Lo-Mendell-Rubin likelihood ratio test; BLRT, bootstrapped likelihood ratio Test; Bold row was the chosen model.

**Figure 1:**
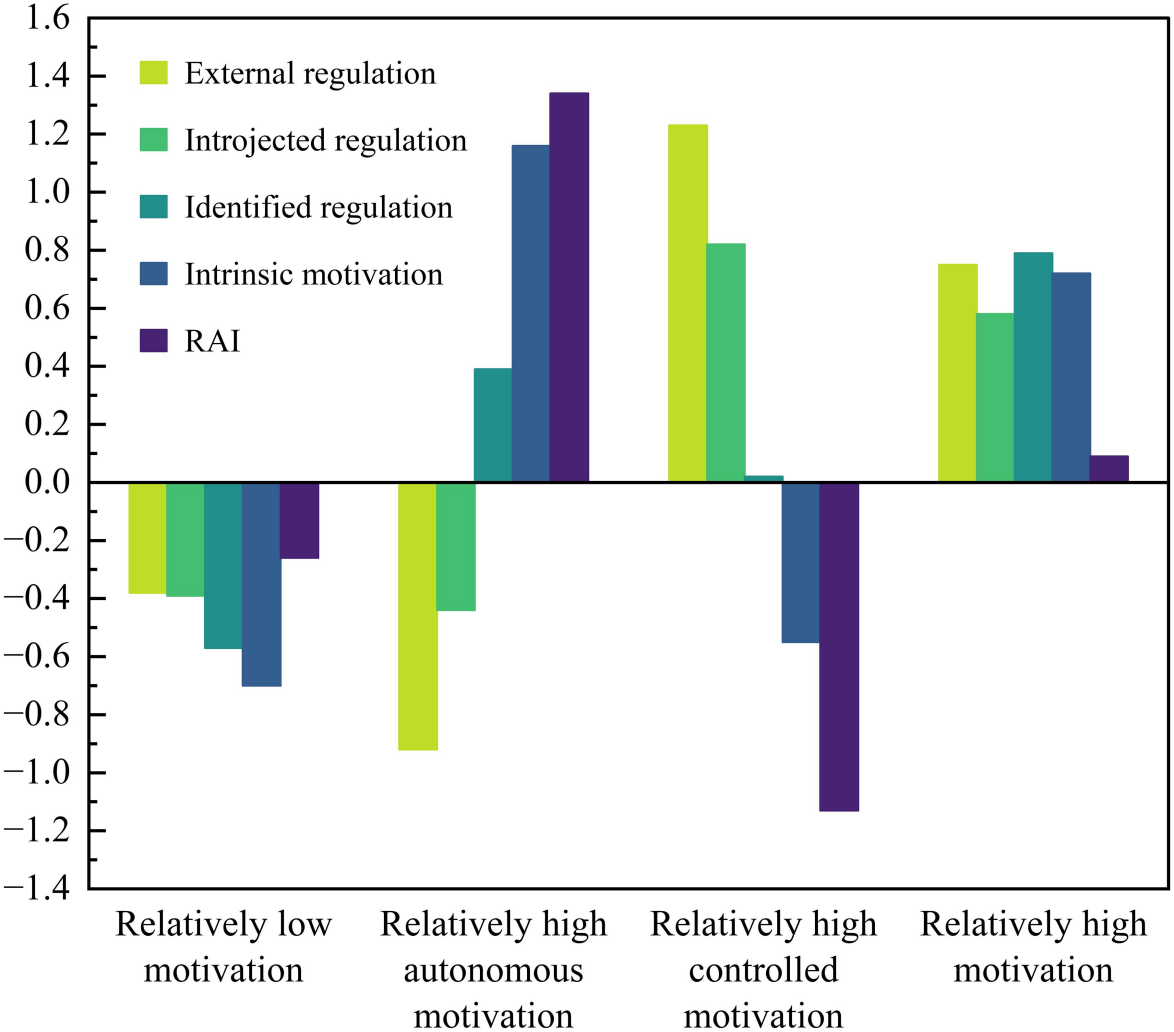
Four classes of academic motivation among master's nursing students based on Z-score means.

In this study, Z-score transformation was first performed on the four dimensions and RAI scores of the SRQ-A. Then, based on the LPA results, the mean Z-scores of the four dimensions and the RAI were calculated for the four latent classes. The mean Z-scores across the four classes are illustrated in Figure 1. Because Z-scores indicate relative positioning within the sample rather than absolute motivational levels, the following labels are interpreted as relative descriptors. Combining the findings in Table 2 and Figure 1, class 1 accounted for 40.3% of the participants, with negative mean values on all four dimensions and the RAI; thus, it was labelled the ‘relatively low motivation’ group. Class 2 made up 23.3% of participants, showing positive values for identified regulation, intrinsic motivation and the RAI, whereas external regulation and introjected regulation yielded negative values. Accordingly, this class was named the ‘relatively high autonomous motivation’ group. Class 3 represented 19.7% of participants, with positive values for external regulation, introjected regulation and identified regulation. Notably, the values of external regulation and introjected regulation were considerably higher than that of identified regulation, while intrinsic motivation and the RAI presented negative values. Therefore, Class 3 was defined as the ‘relatively high controlled motivation’ group. Class 4 accounted for 16.7% of participants, with positive values across all four dimensions and the RAI score; therefore, it was labeled the ‘relatively high motivation’ group.

### 3.4 Associated factors of latent classes of academic motivation

Latent classes of academic motivation among master's nursing students were treated as the dependent variable. Age, gender, nationality, grades, undergraduate training process, nursing as first choice or not, in-service or not, autonomy support, and basic psychological needs were set as independent variables. With the ‘relatively low motivation’ group as the reference group, multinomial logistic regression analysis was performed using the R3STEP method, and the results are presented in Table 3. For each one-year increase in age, the odds of belonging to the ‘relatively high motivation’ group relative to the ‘relatively low motivation’ group were 0.864 times (OR = 0.864, 95% CI = 0.753–0.990), indicating that older age was associated with a lower likelihood of belonging to the ‘relatively high motivation’ group. The odds of females belonging to the ‘relatively high autonomous motivation’ group compared with the ‘relatively low motivation’ group were 3.480 times those of males (OR = 3.480, 95% CI = 1.159–10.450), suggesting that females were more likely to belong to the ‘relatively high autonomous motivation’ group. Students who chose nursing as their first choice had 3.109 times the odds of belonging to the ‘relatively high autonomous motivation’ group (OR = 3.109, 95% CI = 1.529–6.319) and 4.337 times the odds of belonging to the ‘relatively high motivation’ group (OR = 4.337, 95% CI = 1.544–12.182), relative to students who did not choose nursing as their first choice. This finding indicates that students with nursing as their first choice were more likely to fall into the ‘relatively high autonomous motivation’ group and ‘relatively high motivation’ group. For each one-point increase in the autonomy support score, the odds of being in the ‘relatively high autonomous motivation’ and ‘relatively high motivation’ groups were 2.478 times (OR = 2.478, 95% CI = 1.497–4.099) and 2.570 times (OR = 2.570, 95% CI = 1.362–4.850) the original odds, respectively, meaning that higher levels of autonomy support were associated with greater tendency to belong to the ‘relatively high autonomous motivation’ group and ‘relatively high motivation’ group. For each one-point increase in the basic psychological needs score, the odds of belonging to the ‘relatively high autonomous motivation’ group were 1.038 times (OR = 1.038, 95% CI = 1.005–1.074) the original odds, showing that greater satisfaction of basic psychological needs was associated with a greater tendency to belong to the ‘relatively high autonomous motivation’ group. Please refer to the Supplementary Material for the complete regression results (including SE, Wald χ^2^, and exact *p*-values).

**Table 3:** Multinomial logistic regression analysis of latent classes of academic motivation among master's nursing students.

Variable	Class 2 vs. Class 1			Class 3 vs. Class 1			Class 4 vs. Class 1		
	*β*	OR	95% CI	β	OR	95% CI	β	OR	95% CI
Age	−0.094	0.910	0.800–1.036	−0.115	0.892	0.770–1.033	−0.147	0.864*	0.753–0.990
Gender (ref. male)									
Female	1.247	3.480*	1.159–10.450	−0.228	0.796	0.359–1.768	−0.071	0.931	0.371–2.340
Nationality (ref. others)									
Han nationality	0.489	1.630	0.328–8.103	0.074	1.077	0.321–3.616	−0.391	0.676	0.203–2.253
Grades (ref. 2023)									
2021	0.103	1.109	0.450–2.731	0.223	1.250	0.560–2.792	0.085	1.088	0.440–2.693
2022	0.457	1.579	0.824–3.027	−0.051	0.950	0.477–1.894	−0.610	0.543	0.228–1.292
Undergraduate training process (ref. general undergraduate)									
Technical secondary school to undergraduate and college to undergraduate	0.396	1.486	0.787–2.804	−0.643	0.526	0.240–1.151	−0.158	0.854	0.368–1.979
Nursing as first choice (ref. no)									
Yes	1.134	3.109*	1.529–6.319	0.409	1.505	0.779–2.908	1.467	4.337*	1.544–12.182
In-service (ref. no)									
Yes	0.878	2.407	0.631–9.177	−0.033	0.968	0.131–7.143	1.096	2.991	0.655–13.667
Autonomy support	0.907	2.478**	1.497–4.099	0.143	1.154	0.795–1.674	0.944	2.570*	1.362–4.850
Basic psychological needs	0.038	1.038*	1.005–1.074	−0.009	0.991	0.970–1.013	0.023	1.023	0.992–1.056

Class 1, relatively low motivation; Class 2, relatively high autonomous motivation; Class 3, relatively high controlled motivation; Class 4, relatively high motivation. β, unstandardized coefficient; OR, odds ratio; 95% CI, 95% confidence interval. *p < 0.05, **p < 0.001. Please refer to the Supplementary Material for the complete regression results (including SE, Wald χ^2^, and exact *p*-values).

## 4 Discussion

Based on the people-oriented philosophy, this study identifies the heterogeneity of academic motivation among master's nursing students, which can address the limitations of previous variable-centered research and enrich the research on academic motivation among master's nursing students. The results revealed that master's nursing students could be categorized into four classes according to their academic motivation: ‘relatively low motivation’ group, ‘relatively high autonomous motivation’ group, ‘relatively high controlled motivation’ group and ‘relatively high motivation’ group. These classifications were consistent with the theoretical logic of the four motivational classes (a good quality motivation group, a poor quality motivation group, a low quantity motivation group, and a high quantity motivation group) divided by Vansteenkiste et al. (16) based on SDT. The ‘relatively low motivation’ group (40.3%) exhibited relatively low levels of both controlled motivation and autonomous motivation, with an overall relatively low level of motivation. The ‘relatively high autonomous motivation’ group (23.3%) was characterized by low controlled motivation and high autonomous motivation, reflecting a relatively high quality of motivation. The ‘relatively high controlled motivation’ group (19.7%) showed high controlled motivation and low autonomous motivation, reflecting a relatively low quality of motivation. The ‘relatively high motivation’ group (16.7%) exhibited relatively high levels of both controlled motivation and autonomous motivation, with an overall relatively high level of motivation. Research has shown that among students categorized into the four classes of the ‘relatively low motivation’ group, ‘relatively high autonomous motivation’ group, ‘relatively high controlled motivation’ group, and ‘relatively high motivation’ group, those in the ‘relatively high autonomous motivation’ group tend to exhibit stronger determination, better self-regulation ability, and superior learning outcomes, whereas controlled motivation is positively correlated with test anxiety and procrastination regardless of the level of individual autonomous motivation (16). In this study, the ‘relatively high autonomous motivation’ group accounted for only 23.3% of the sample. This may be attributed to the fact that some students have insufficient research foundation, fail to adjust to intensive postgraduate academic training, and are prone to developing fear of difficulties (14). Furthermore, master's nursing students must balance multiple demands of coursework, research, and clinical practice, often engaging selectively in learning tasks and demonstrating relatively low autonomy (15). Educators should provide tiered interventions for different classes of students. For the ‘relatively low motivation’ group students, educators should strengthen their recognition of professional value, deliver individualized support, and focus on fostering their intrinsic interest and academic confidence. For the ‘relatively high autonomous motivation’ group students, educators should consolidate their strengths, build an educational environment supportive of intrinsic motivation development, and attend to the maintenance of their motivation quality and learning outcomes. For the ‘relatively high controlled motivation’ group students, educators should reinforce the meaning of learning and implement psychological adjustment training to support their transformation of external pressure into intrinsic motivation. For the ‘relatively high motivation’ group students, educators should preserve their enthusiasm for learning, reduce reliance on external evaluation, helping their motivational structure develop toward a more autonomous orientation.

The results of this study indicate that among sociodemographic characteristics, age, gender and nursing as first choice are associated factors for the latent classes of academic motivation among master's nursing students. Older age was associated with a lower likelihood of belonging to the ‘relatively high motivation’ group. Abdi et al. (43) found that older students often perceive an age gap with younger peers and various constraints during further study, and tend to report relatively lower levels of academic motivation. Compared with males, females are more likely to belong to the ‘relatively high autonomous motivation’ group. This is consistent with the results reported by Wang et al. (44), who indicated that females possess higher levels of autonomous motivation. Nursing is a female-dominated profession, most females choose this major out of professional identity and pursuit of values (45). Their learning behaviors are primarily driven by internal factors such as personal interest and career aspirations, and their level of autonomous motivation is relatively high. Compared with the ‘relatively low motivation’ group, students with nursing as their first choice were more likely to be classified into the ‘relatively high autonomous motivation’ group and the ‘relatively high motivation’ group. Among these students, some had an intrinsic identification with and interest in the nursing profession, exhibited a high level of autonomous motivation, and showed self-driven characteristics in their learning behavior; others valued the employment prospects of the nursing profession while also possessing a strong desire for knowledge, maintaining relatively high levels of both controlled motivation and autonomous motivation (46). It is suggested that nursing educators focus on the psychological adjustment of male and older students, and provide career planning education for students who did not chose nursing as their first choice, with attention to stimulating and maintaining their autonomous motivation.

Autonomy support is associated with the latent classes of academic motivation among master's nursing students. Compared with the ‘relatively low motivation’ group, master's nursing students who perceive higher levels of autonomy support have a greater probability of belonging to the ‘relatively high autonomous motivation’ group and ‘relatively high motivation’ group. This study identified an association between autonomy support and the ‘relatively high autonomous motivation’ group. This finding aligns with studies by Johansen et al. (47) and Vaquero-Solís et al. (48), which reported a positive association between perceived autonomy support and autonomous motivation. Torbergsen et al. (49) noted that autonomy-supportive teaching behaviors, such as offering students choices in learning and respecting their personal perspectives, are positively associated with students' learning initiative, classroom engagement, intrinsic academic motivation and academic performance. Conversely, Johansen et al. (47) found that teaching practices that disregard students' needs and adopt controlling styles are negatively associated with students' internal acceptance and levels of autonomous motivation. This study further identified an association between autonomy support and the ‘relatively high motivation’ group. Master's nursing students are required to cope with multiple external demands including research and clinical work. Autonomy support does not eliminate these demands; rather, it is associated with the internalization of such demands, during which controlled motivation coexists with autonomous motivation. Against the backdrop of a global shortage of nursing workforce and growing demands for nursing education, the implementation of autonomy-supportive teaching strategies bears important practical implications for improving the quality of nursing talent cultivation. Nursing educators are recommended to fully respect students' principal role, foster a supportive teacher–student interaction atmosphere, adopt guiding rather than commanding communication styles, and attach importance to the internalization of academic motivation among master's nursing students.

Basic psychological needs are associated with the latent classes of academic motivation among master's nursing students. Compared with the ‘relatively low motivation’ group, individuals with higher satisfaction of basic psychological needs are more likely to belong to the ‘relatively high autonomous motivation’ group. As reported by Orsini et al. (50), satisfaction of basic psychological needs is positively correlated with individuals engaging in activities based on autonomous motivation. Adarlo et al. (51) illustrated that during postgraduate training, satisfaction of the need for autonomy is reflected in supervisors' respect for students' research interests and encouragement of independent thinking; satisfaction of the need for competence manifests as supervisors providing positive feedback, inspiring critical thinking, and offering practical opportunities; satisfaction of the need for relatedness means supervisors deliver emotional support, maintain understanding and empathy, and build mutually respectful and trusting supervisor–student relationships. When these needs are met, students are more likely to exhibit higher levels of autonomous motivation and engage with academic tasks in a more proactive manner (52). Conversely, previous research has indicated that frustration of psychological needs is associated with higher burnout levels and stronger dropout intentions among students (53). Educators should build a supportive academic community, respect students' personal development aspirations, ensure the satisfaction of their basic psychological needs.

## 5 Limitations

This study has several limitations. First, the cross-sectional design precludes examination of the temporal stability of latent classes and the dynamic trajectories of academic motivation. Longitudinal designs are recommended for future research to further explore their evolutionary mechanisms. Second, convenience sampling may introduce selection bias. The sample comprised a high proportion of female (86.2%), Han nationality (93.3%), and not in-service students (91.8%). Caution should be exercised when generalizing the findings to male, others nationality, and in-service master's nursing students. In addition, the sample was recruited from multiple universities, with uneven sample sizes across universities. Since the exact number of responses from each university could not be traced, the statistical correction for the clustering effect by university was not performed. This may lead to underestimated standard errors, potentially confounding institution-level and individual-level factors and introducing bias in the interpretation of individual-level associated factors. Meanwhile, the examination of inter-institutional heterogeneity was limited. Future studies may adopt stratified random sampling or multicenter cluster sampling designs, together with appropriate correction using cluster-robust standard errors, to more accurately estimate inter-institutional differences and their associations with latent classes of academic motivation. Third, data were collected via self-reported questionnaires, which may lead to common method variance. Multi-source data collection combined with objective indicators is suggested in subsequent studies. Fourth, mixed survey administration modes may induce bias in measurement consistency. Future research should adopt a unified administration mode whenever possible to eliminate this confounding interference. Fifth, the SRQ-A, LCQ and BPNSS were originally validated primarily among middle school students. Although the Cronbach's alpha coefficients in the present sample were acceptable, the CFA results indicated that only the SRMR of the LCQ met the recommended criterion, while none of the fit indices for the SRQ-A and BPNSS reached the recommended thresholds, suggesting that the original factor structures provided a poor fit to the present sample. Although the LPA was based on the 30 item scores of the SRQ-A rather than dimension scores, these items were the direct basis for identifying latent classes, and the instability of the factor structure may still affect the interpretation of the substantive meaning of the latent classes. Therefore, the related conclusions should be interpreted with caution. Future research should develop or revise measurement instruments specifically for master's nursing students and further examine the stability and generalizability of the latent classes. Sixth, external criterion variables were not included to examine the validity of latent classes. Future research should incorporate external outcome variables for validation.

## 6 Conclusion

In summary, this study found that the classification characteristics of academic motivation among master's nursing students were obvious and classified them into four classes: ‘relatively low motivation’ group, ‘relatively high autonomous motivation’ group, ‘relatively high controlled motivation’ group, and ‘relatively high motivation’ group. Age, gender, nursing as first choice, autonomy support, and basic psychological needs were associated factors of the different classes. These findings suggest that autonomy-supportive learning environments and attention to students' basic psychological needs may represent relevant areas for educational support and future intervention research.

## Data Availability

The raw data supporting the conclusions of this article will be made available by the authors, without undue reservation.
