# Supplementary material for: Latent profile analysis and associated factors of academic motivation among master's nursing students: a cross-sectional study
Source: Front Med (Lausanne). 2026 Oct 14;13:1962934. doi: 10.3389/fmed.2026.1962934 (PMC13713825; doi:10.3389/fmed.2026.1962934)
Supplement: Supplementary file 1 [file Supplementaryfile1.docx]

**Supplementary Figure S1. Three classes of academic motivation among master’s nursing students based on Z-score means**

**Supplementary Figure S2. Five classes of academic motivation among master’s nursing students based on Z-score means**

**Supplementary Table S1. Multinomial logistic regression analysis of latent classes of academic motivation among master’s nursing students（*N*=390）**

| **Variable** | **β** | **SE** | **Wald χ²** | ***P*** | **OR** | **95% CI** |
| --- | --- | --- | --- | --- | --- | --- |
| **Class 2 vs. Class 1** |  |  |  |  |  |  |
| Age (years) | -0.094 | 0.066 | 2.074 | 0.150 | 0.910 | 0.800-1.036 |
| Gender (ref. male) |  |  |  |  |  |  |
| Female | 1.247 | 0.561 | 4.946 | 0.026 | 3.480 | 1.159-10.450 |
| Nationality (ref. others) |  |  |  |  |  |  |
| Han nationality | 0.489 | 0.818 | 0.358 | 0.550 | 1.630 | 0.328-8.103 |
| Grades (ref. 2023) |  |  |  |  |  |  |
| 2021 | 0.103 | 0.460 | 0.050 | 0.823 | 1.109 | 0.450-2.731 |
| 2022 | 0.457 | 0.332 | 1.893 | 0.169 | 1.579 | 0.824-3.027 |
| Undergraduate training process (ref. general undergraduate) |  |  |  |  |  |  |
| Technical secondary school to undergraduate and college to undergraduate | 0.396 | 0.324 | 1.491 | 0.222 | 1.486 | 0.787-2.804 |
| Nursing as first choice (ref. no) |  |  |  |  |  |  |
| Yes | 1.134 | 0.362 | 9.797 | 0.002 | 3.109 | 1.529-6.319 |
| In-service (ref. no) |  |  |  |  |  |  |
| Yes | 0.878 | 0.683 | 1.654 | 0.199 | 2.407 | 0.631-9.177 |
| Autonomy support | 0.907 | 0.257 | 12.482 | 0.000 | 2.478 | 1.497-4.099 |
| Basic psychological needs | 0.038 | 0.017 | 4.858 | 0.027 | 1.038 | 1.005-1.074 |
| **Class 3 vs. Class 1** |  |  |  |  |  |  |
| Age (years) | -0.115 | 0.075 | 2.320 | 0.128 | 0.892 | 0.770-1.033 |
| Gender (ref. male) |  |  |  |  |  |  |
| Female | -0.228 | 0.407 | 0.315 | 0.575 | 0.796 | 0.359-1.768 |
| Nationality (ref. others) |  |  |  |  |  |  |
| Han nationality | 0.074 | 0.618 | 0.014 | 0.904 | 1.077 | 0.321-3.616 |
| Grades (ref. 2023) |  |  |  |  |  |  |
| 2021 | 0.223 | 0.410 | 0.297 | 0.586 | 1.250 | 0.560-2.792 |
| 2022 | -0.051 | 0.352 | 0.021 | 0.885 | 0.950 | 0.477-1.894 |
| Undergraduate training process (ref. general undergraduate) |  |  |  |  |  |  |
| Technical secondary school to undergraduate and college to undergraduate | -0.643 | 0.400 | 2.579 | 0.108 | 0.526 | 0.240-1.151 |
| Nursing as first choice (ref. no) |  |  |  |  |  |  |
| Yes | 0.409 | 0.336 | 1.479 | 0.224 | 1.505 | 0.779-2.908 |
| In-service (ref. no) |  |  |  |  |  |  |
| Yes | -0.033 | 1.020 | 0.001 | 0.974 | 0.968 | 0.131-7.143 |
| Autonomy support | 0.143 | 0.190 | 0.564 | 0.453 | 1.154 | 0.795-1.674 |
| Basic psychological needs | -0.009 | 0.011 | 0.579 | 0.447 | 0.991 | 0.970-1.013 |
| **Class 4 vs. Class 1** |  |  |  |  |  |  |
| Age (years) | -0.147 | 0.070 | 4.414 | 0.036 | 0.864 | 0.753-0.990 |
| Gender (ref. male) |  |  |  |  |  |  |
| Female | -0.071 | 0.470 | 0.023 | 0.880 | 0.931 | 0.371-2.340 |
| Nationality (ref. others) |  |  |  |  |  |  |
| Han nationality | -0.391 | 0.614 | 0.404 | 0.525 | 0.676 | 0.203-2.253 |
| Grades (ref. 2023) |  |  |  |  |  |  |
| 2021 | 0.085 | 0.462 | 0.033 | 0.855 | 1.088 | 0.440-2.693 |
| 2022 | -0.610 | 0.442 | 1.910 | 0.167 | 0.543 | 0.228-1.292 |
| Undergraduate training process (ref. general undergraduate) |  |  |  |  |  |  |
| Technical secondary school to undergraduate and college to undergraduate | -0.158 | 0.429 | 0.135 | 0.714 | 0.854 | 0.368-1.979 |
| Nursing as first choice (ref. no) |  |  |  |  |  |  |
| Yes | 1.467 | 0.527 | 7.756 | 0.005 | 4.337 | 1.544-12.182 |
| In-service (ref. no) |  |  |  |  |  |  |
| Yes | 1.096 | 0.775 | 1.999 | 0.157 | 2.991 | 0.655-13.667 |
| Autonomy support | 0.944 | 0.324 | 8.486 | 0.004 | 2.570 | 1.362-4.850 |
| Basic psychological needs | 0.023 | 0.016 | 2.111 | 0.146 | 1.023 | 0.992-1.056 |

Note: Class 1, relatively low motivation; Class 2, relatively high autonomous motivation; Class 3, relatively high controlled motivation; Class 4, relatively high motivation.

β, unstandardized coefficient; SE, standard error; OR, odds ratio; 95% CI, 95% confidence interval.
